# Prognostic Significance of Serum Lactic Acid, Lactate Dehydrogenase, and Albumin Levels in Patients with Metastatic Colorectal Cancer

**DOI:** 10.1155/2018/1804086

**Published:** 2018-12-09

**Authors:** Yan Wei, Hui Xu, Jing Dai, Jin Peng, Wenbo Wang, Ling Xia, Fuxiang Zhou

**Affiliations:** ^1^Department of Radiation and Medical Oncology, Zhongnan Hospital, Wuhan University, Wuhan 430071, China; ^2^Hubei Key Laboratory of Tumor Biological Behaviors, Zhongnan Hospital, Wuhan University, Wuhan 430071, China; ^3^Hubei Clinical Cancer Study Centre, Zhongnan Hospital, Wuhan University, Wuhan 430071, China

## Abstract

**Aim:**

To identify the population of patients with high risk of distant metastasis and the poor prognosis before treatment, so as to provide early intervention and better treatment decision.

**Method:**

69 patients with nonmetastatic colorectal cancer (non-mCRC) and 57 with metastatic CRC (mCRC) were enrolled to evaluate the prognostic value of serum albumin (ALB), serum lactate (SLA), and lactate dehydrogenase (LDH) in patients with metastatic CRC. We then followed up the 57 patients with mCRC. The T test, Chi square test, Kaplan-Meier survival analysis model, and Multivariate Cox proportional hazards regression model were applied to assess the prognostic significance of SLA, LDH, and serum ALB on the patients with mCRC.

**Results:**

Compared with the non-mCRC group, the patients with mCRC had an elevated level of blood lactate (*P=0.01*) and LDH (*P<0.01*) and a reduced level of ALB (*P<0.01*). Multivariable analysis showed the elevated LDH combined with elevated SLA (HR=2.922, 95%CI=0.971-8.793, P=0.056), the reduced ALB (HR=0.417, 95%CI=0.230-0.754, P=0.004), and the elevated CA199 (HR=2.072, 95%CI=1.125-3.816, P=0.019) were independent prognostic factors for PFS of patients with mCRC. The elevated LDH (HR=2.204, 95%CI=1.000-4.858, P=0.050), reduced ALB (HR=0.459, 95%CI=0.236-0.892, P=0.022), elevated LDH combined with elevated SLA (HR=3.187, 95%CI=1.019-9.970, P=0.046), and the primary site of tumor (HR=0.359, 95%CI=0.174-0.740, P=0.006) were independent prognostic factors for OS of patients with mCRC.

**Conclusions:**

Taken together, our results implicate that the elevated LDH combined with elevated SLA and the reduced ALB are prognostic indicators for patients with mCRC.

## 1. Introduction

According to the Global Cancer Statistics in 2012, colorectal cancer (CRC) is the second most commonly diagnosed cancer in females and the third in males in the world [1]. It is one of the most malignant tumors with high morbidity and poor prognosis [2]. Distant metastasis is the main cause of the treatment failure of the patients with colorectal cancer. Therefore, it is necessary to identify the population of patients with high risk of distant metastasis and the poor prognosis before treatment, so as to provide early intervention and better treatment decision.

In recent years, the intensive study of biomarkers has contributed to the early diagnosis, treatment, and prognosis of CRC. Prognostic biomarkers can estimate the natural course of the disease and divide the tumor into two groups: good prognosis and poor prognosis [3, 4]. They are involved in different processes, such as cellular proliferation, differentiation, angiogenesis, invasion, and metastasis [5, 6]. To discover the potential biomarkers, which could be detected in blood through noninvasive methods, it is important to study the pathological basis of CRC [7].

Serum albumin is closely correlated with the degree of malnutrition and is a regularly used marker of nutrition status [8]. An ongoing tumor related systemic inflammatory response may also contribute to the progressive loss of albumin [9]. Therefore, it may be a valuable prognostic factor for poor survival in CRC patients.

The metabolism of tumor cells has its own characteristics. To cooperate with the rapid development of tumor, the tumor cells produce energy through glycolysis, which is called the Warburg effect [10]. This change in tumor cell metabolism is considered as a major change in tumor transformation. Hypoxic microenvironment is another feature of malignant tumors [11]. In hypoxic conditions, LDH can convert pyruvate to lactic acid to support tumor cells [12]. Hypoxia inducible factor 1*α* (HIF-1*α*) can transcriptionally upregulate LDH-A in tumor cells to ensure anaerobic glycolysis and produce enough lactate. High levels of lactate can in turn promote higher expression of HIF-1*α*. Therefore, HIF-1*α* and LDH-A can regulate each other and reinforce each other's expression. [13]. In addition, high LDH is closely related to increased tumor vascular density and decreased lymphocyte infiltration. Therefore, the elevated levels of SLA and LDH indicate the aggravated tumor burden, tumor angiogenesis, tumor progression, and poor prognosis of patients [14]. However, there is no clear clinical evidence of the relationship between serum ALB, SLA, and LDH and the prognosis of mCRC patients.

A total of 126 patients were collected in this study, including 57 metastatic and 69 nonmetastatic CRC patients. We compared the level of serum ALB, SLA, and LDH measured before the first-line chemotherapy in patients with metastatic colorectal cancer and before adjuvant chemotherapy in patients with nonmetastatic cancer. We then followed up the 57 metastatic CRC patients to evaluate the effect of serum ALB, SLA, and LDH levels on the prognosis.

## 2. Materials and Methods

### 2.1. Patients Selection

A retrospective study was performed on 126 patients diagnosed with colorectal cancer between January 2013 and December 2016 in the Department of Radiation and Medical Oncology of Zhongnan Hospital of Wuhan University in Hubei. Those patients included 57 patients with mCRC and 69 patients with non-mCRC. This study was reviewed and approved by the Ethics Committee of Zhongnan Hospital (Scientific Ethical Approval No. 2018005). Inclusion criteria were as follows: (1) All 126 patients were confirmed histologically as colorectal adenocarcinoma. (2) The patients with non-mCRC received standard postoperative adjuvant therapy and patients with mCRC received standard first-line chemotherapy. (3) The metastasis was confirmed by histopathology, computerized tomographic scanning (CT), magnetic resonance imaging (MRI), or positron emission tomography (PET). (4) All patients had the vital information available for analysis, such as age, gender, pathological type, chemotherapy regiments, and the level of SLA, LDH, ALB, total protein (TP), carcinoembryonic antigen (CEA), and carbohydrate antigen 199 (CA199). (5) All patients had an Eastern Cooperative Oncology Group (ECOG) score of less than 2. (6) The blood routine parameters of all patients were within the normal range: WBC≥3.5 × 10^9^/L, Hb≥90g/L, PLT≥100 × 10^9^/L. (7) The normal liver and renal function examination: the ALT and AST is less than 2.5 times of the normal value; the serum creatinine is less than 1.5 times of the normal value. The exclusion criteria included (1) patients with hypoxemia; (2) patients taking metformin; (3) patients who have had epilepsy during hospitalization; (4) patients with severe hepatic and renal dysfunction; and (5) patients with severe heart disease, such as uncontrolled hypertension, congestive heart failure, arrhythmia, and coronary heart disease.

### 2.2. Data Collection

We collected the data of SLA, LDH, albumin, total protein, CEA, CA199, and the degree of pathological differentiation which were measured within 1 week before the first chemotherapy. All serological indexes were measured by standard method. Low serum lactate level was defined as less than 4.0mmol/L, and elevated serum lactate level was defined as ≥4.0mmol/L. Normal serum LDH was defined as 100 to 225U/L and elevated LDH was defined as ≥225U/L (Butt, Michaels and Kissinger 2002). Patients with albumin level <40g/L and total protein <70g/L were classified as the reduced ALB group and reduced TP group, respectively. Elevated CEA was defined as ≥5ng/ml and elevated CA199 as ≥40U/ml.

### 2.3. Patients Follow-Up

We followed up the 57 metastatic CRC patients. The primary end points of this study were PFS and OS. The last follow-up time was April 19, 2018. OS time was defined as the time from the date of the initiation of first-line chemotherapy to death or the last follow-up visit if the patient was still alive by the end of the study. The PFS time was from the first-line chemotherapy to the time of tumor progression. If the patient was still alive at the end of the study and diagnosed without progression, the PFS time was the time of the last follow-up. Patients were followed up regularly by phone call. The progression of the disease was defined as the finding of the metastasis by imaging examination (CT, MRI, PET/CT, ultrasound, etc.), endoscopic or pathological examination.

### 2.4. Statistical Methods

Double entry was applied to ensure the accuracy of data. All data were statistically analyzed by the Statistical Package for the Social Sciences version 20.0 software package (SPSS20.0). Descriptive statistics were used to illustrate the general characteristic of the patients. The Chi square test and T test were used to analyze the general information of the patients. The univariate and multivariate analyses were used to identify factors that influenced the survival time of patients. Multivariate analysis was performed with the Cox proportional hazards model to test independent significance while adjusting for covariates. Data was presented as hazard ratios (HRs) and 95% confidence intervals (CIs). The Kaplan-Meier method was used to calculate the survival curve. P value<0.05 was considered significant.

## 3. Results

### 3.1. Patients' Characteristics

A total of 126 patients were enrolled in this study, including 69 nonmetastatic and 57 metastatic colorectal cancer patients. As shown in Table 1, there were no significant differences in gender, age, and the location of tumor between the two groups.

### 3.2. The Levels of SLA, LDH, and ALB Are Elevated in mCRC Patients

**Figure 1:**
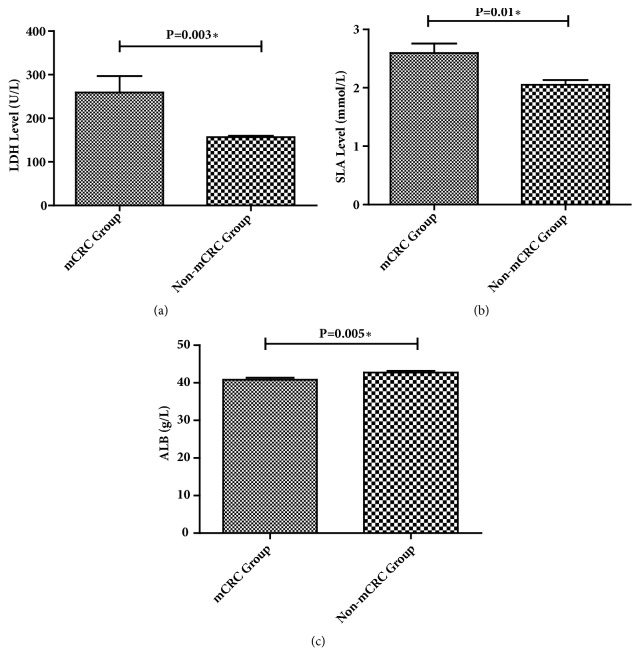
Comparison of LDH, SLA, and ALB levels in patients with mCRC and Non-mCRC before the chemotherapy (N=126). *∗* We define P < 0.05 as statistical difference. T test and nonparametric test were used to compare the LDH, SLA, and ALB levels of the two groups.

We compared the levels of SLA, LDH, and serum albumin measured before the first-line chemotherapy in patients with mCRC and before adjuvant chemotherapy in patients with non-mCRC. The SLA (P=0.01) and LDH (P<0.01) levels in the metastatic group were higher than those in the nonmetastatic group, and ALB (P<0.01) level in the metastatic group was lower than that in the nonmetastatic group (Figure 1). Therefore, SLA, LDH, and albumin can be used as a marker of metastasis in patients with colorectal cancer.

### 3.3. Univariate Survival Analysis of Patients with mCRC

We further followed up the 57 metastatic patients. The median follow-up time was 21.6 months. The median overall survival (OS) of the mCRC patients was 19.9 months and the median progression-free survival (PFS) was 10.8 months. All the metastatic patients were pathologically classified as stage IV. Of the metastatic patients, 30 (52.6%) cases were colon cancer and 27 (47.4%) cases were rectal cancer. 36 patients were treated with mFOLFOX6 and 21 patients with FOLFIRI chemotherapy.

We divided the patients into two groups according to the level of SLA, LDH, and ALB. The basic information of different groups of patients was compared. The general information (sex and age) of the patient showed no significant difference between the two groups (Table 2). While the level of LDH may be related to the degree of tumor differentiation (P=0.036), and the level of ALB may be related to the degree of differentiation (P=0.004) and the primary location of the tumor (P=0.019).

**Figure 2:**
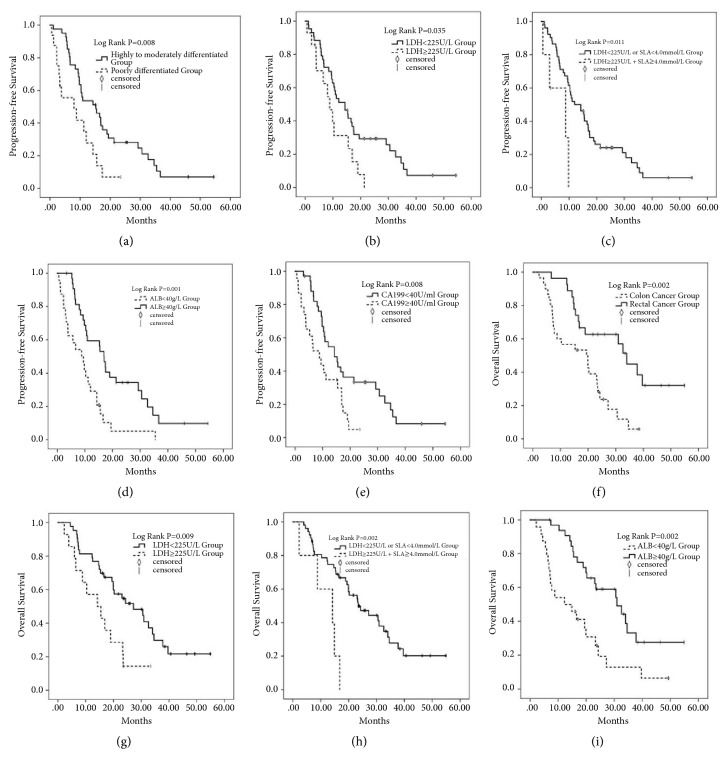
The Kaplan-Meier statistical analysis was used to analyze the survival curves of patients with different LDH, ALB, SLA, CA199, and primary tumor sites. (a) The progression-free survival curve of the patients with different degree of differentiation. (b) The progression-free survival curve of the patients with different LDH. (c) The progression-free survival curve of the patients with different LDH combined with SLA. (d) Progression-free survival curve of patients with different ALB. (e) The progression-free survival curve of the patients with different CA199. (f) Total survival time curve of patients with different tumor location. (g) The total survival time curve of the patients with different LDH. (h) The total survival time curve of the patients with different LDH combined with SLA. (i) The total survival time curve of the patients with different ALB.

Univariate survival analysis method was used to analyze the sex, age, primary site of tumor, chemotherapy regimen, blood lactate level, lactate dehydrogenase level, serum albumin level, total protein level, CEA, and CA199 of mCRC patients. In univariate analysis (Table 3), the location of tumor (P = 0.034), low degree of pathological differentiation (P = 0.010), CA199≥40U/ml (P = 0.009), serum albumin<40g/L (P = 0.002), LDH≥225 U/L (P = 0.038), and LDH≥225 U/L combined with SLA≥4.0 mmol/L (P = 0.018) were identified as poor prognostic factors for the PFS of patients with mCRC. The primary tumor occurred in colon (P = 0.002), LDH≥225U/L (P = 0.011), ALB<40g/L (P = 0.003), and low degree of pathological differentiation (P = 0.028) were poor prognostic factors for OS of patients with mCRC. And the Kaplan-Meier statistical analysis was used to analyze the survival curves of patients with different LDH, ALB, SLA, CA199, and primary tumor sites (Figure 2).

### 3.4. Multivariate Analysis of Patients with mCRC

**Figure 3:**
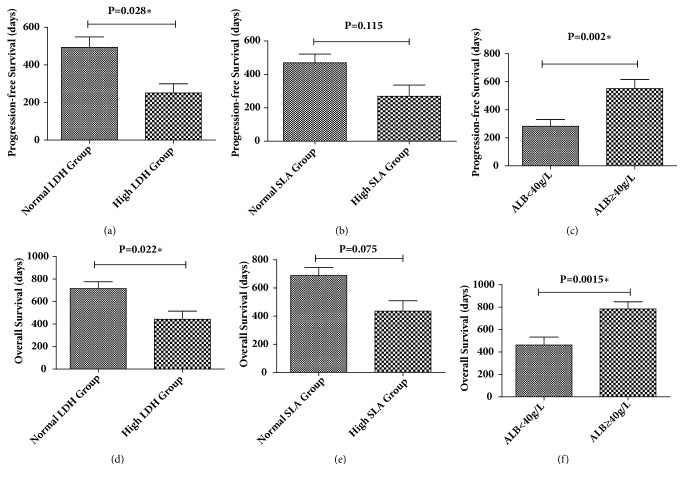
The progression-free survival time and overall survival time of patients with mCRC grouped by the levels of LDH, SLA, and ALB. The independent sample T test and nonparametric test were used to analyze the progression-free survival time and overall survival time of patients with mCRC grouped by the levels of LDH, SLA, and ALB. *∗* We define P < 0.05 as statistical difference.

We used multivariate analysis to further analyze the factors that are significant in univariate analysis, including the primary site of the tumor, the degree of differentiation, the levels of SLA, LDH, ALB, and CA199. The reduced ALB (HR=0.417, 95%CI=0.230-0.754, P=0.004), elevated CA199 (HR=2.072, 95%CI=1.125-3.816, P=0.019), and the elevated LDH combined with elevated SLA (HR=2.922, 95%CI=0.971-8.793, P=0.056) were independent prognostic factors for PFS of mCRC patients. The primary site of tumor (HR=0.359, 95%CI=0.174-0.740, P=0.006), the elevated LDH (HR=2.204, 95%CI=1.000-4.858, P=0.050), the reduced ALB (HR=0.459, 95%CI=0.236-0.892, P=0.022), and the elevated LDH combined with elevated SLA (HR=3.187, 95%CI=1.019-9.970, P=0.046) were independent influence factors for OS of mCRC patients (Table 4). The COX regression model was used for statistical analysis. PFS time and OS time in normal baseline LDH group (P = 0.028 for PFS, P = 0.022 for OS), normal baseline SLA (P = 0.115 for PFS, P = 0.075 for OS), and ALB≥40g/L group (P = 0.002 for PFS, P = 0.0015 for OS) were longer compared with high baseline LDH group, high baseline group, and ALB<40g/L group (Figure 3).

## 4. Discussion

There are many factors that lead to a reduction in the level of albumin: the capillary leakage into the interstitium and the acceleration of degradation and the reduction of synthesis caused by inflammation [15, 16]. The prognostic value of the combination of an elevated CRP concentration and hypoalbuminaemia was verified in many cancers including colorectal cancer [15]. The preoperative hypoalbuminemia was known to be a strong predictor of poor outcomes after gastrointestinal surgery [17, 18]. However, the relationship between hypoalbuminemia and the prognosis of mCRC patients after chemotherapy is not clear.

The present study demonstrated that the hypoalbuminemia was an independent prognostic factor for metastatic colorectal cancer patients. 24 (42.1%) of the 57 metastatic patients were hypoalbuminemic. The univariate and multivariate analyses showed that the hypoalbuminemia was significantly associated with postchemotherapy morbidity.

We also found that the metastatic group had a higher level of LDH and SLA than the nonmetastatic group. According to the univariate and multivariate analysis, the elevated LDH level was identified as poor prognostic factors for OS and PFS of mCRC patients. Compared with patients with normal baseline SLA and LDH, patients with SLA≥4.0 mmol/L combined with LDH≥225mmol/L had a s 3.1-fold risk of cancer death (P=0.046, HR=3.187, 95%CI=1.019-9.970).

Many studies have found that the accumulation of LDH might play a role in the development of a variety of tumors such as breast cancer, nasopharyngeal carcinoma, malignant melanoma, and pancreatic cancer [19–22]. It was found that the high concentration of lactic acid could inhibit monocyte migration and cytokines release, increase the activity of arginase I, and activate the IL-23/IL-17 pathway. Lactic acid could also inhibit the proliferation of CD8+T cells, reduce the secretion of cytokine IFN-*γ*, and inhibit the activity of NK cells, thus resulting in the immune escape of the tumor cells [23–26].

Intracellular lactic acid could inhibit the degradation of hypoxia inducible factor -1 alpha (HIF-1*α*) in endothelial cells under no hypoxia state and significantly increase VEGF and fibroblast growth factors produced by endothelial cells thus promoting tumor angiogenesis [27, 28]. In addition, lactic acid could be transported to cells through MCTs, which makes DNA repair gene upregulate and improves the survival of tumor cells after chemotherapy, leading to tumor cell resistance to chemotherapeutic drugs [29]. Therefore, the accumulation of lactic acid promoted the formation of tumor vessels and the metastasis of tumor and mediated immune escape. This is in line with the results of our study.

Moreover, we also found that the elevated CA199 level (*P=0.031*) and the tumor location in the colon (P=0.006) were identified as poor prognostic factors. This is in line with the results of a number of previous studies [30].

Taken together, the levels of ALB, LDH, SLA, and CA199 measured before first chemotherapy in patients with metastatic colorectal cancer may serve as a marker of deterioration and chemoresistance. The weakness of this study is the small number of cases and the short follow-up time. Therefore, an independent validation study by multicenter groups is needed for the translation of these discoveries to clinical useful strategies.

## Figures and Tables

**Table 1:** General information comparison between patients with mCRC and Non-mCRC (N=126).

**Contents**	**Sex**		**Age**	**The location of tumor**		**Pathological type**	
	**Male**	**Female**	**(years)**	**Colon**	**Rectum**	**Poorly**	**Well**
**mCRC Group (57)**	36	21	55.3±11.2	30	27	16	41
**Non-mCRC Group (69)**	39	30	54.2±11.6	29	40	8	61
**X** ^**2**^ **/t**	t=0.571		t=0.255	t=1.409		t=5.495	
**P Value**	P=0.450		P=0.593	P=0.235		**P=0.019** **∗**	

*∗* We define P < 0.05 as statistical difference.

*∗*Chi square test and T test were used to analyze the basic information of the two groups.

**Table 2:** Baseline characteristics of patients with mCRC grouped by the levels of LDH, SLA, and ALB.

**Contents**		**Sex**		**Age (year)**		**Locations (cancer)**		**Pathological type**		**Other treatment before chemotherapy**	
		**Male**	**Female**	**<56**	**≥56**	**Rectum**	**Colon**	**Poor**	**Well**	**Yes**	**No**
**LDH**	**LDH≥225**	9	5	4	10	6	8	7	7	2	12
	**LDH<225**	27	16	21	22	21	22	9	34	12	31
	X^2^**/t**	0.010		1.035		0.151		4.420		0.450	
	**P Value**	0.920		0.309		0.697		**0.036** **∗**		0.502	

**SLA**	**SLA≥4.0**	6	3	5	4	4	5	4	5	1	8
	**SLA<4.0**	30	18	20	28	23	25	12	36	13	35
	X^2^**/t**	0.000		0.589		0.000		0.620		0.360	
	**P Value**	1.000		0.433		1.000		0.431		0.549	

**ALB**	**ALB≥40**	22	11	14	19	20	13	4	29	9	24
	**ALB<40**	14	10	11	13	7	17	12	12	5	19
	X^2^**/t**	0.415		0.066		5.509		8.087		0.311	
	**P Value**	0.520		0.798		**0.019** **∗**		**0.004** **∗**		0.577	

Using chi square test, we estimate the baseline characteristics of patients with mCRC grouped by the levels of LDH, SLA, and ALB.

*∗* We define P < 0.05 as statistical difference.

**Table 3:** Univariate analysis of patients with metastatic colorectal cancer (N=57).

**Prognostic factors**	**PFS**			**OS**		
	**HR**	**95**%**CI**	**P value**	**HR**	**95**%**CI**	**P value**
**Age (56y)**	0.892	0.499-1.596	0.701	0.945	0.504-1.794	0.861
**Sex (Female)**	0.961	0.538-1.717	0.894	0.763	0.404-1.442	0.405
**Locations (Rectum)**	0.530	0.294-0.953	**0.034** **∗**	0.351	0.178-0.690	**0.002** **∗**
**Pathological type (Poorly) **	2.340	1.223-4.474	**0.010** **∗**	2.113	1.083-4.124	**0.028** **∗**
**Other treatment **	0.715	0.368-1.387	0.320	0.952	0.465-1.952	0.894
**Regimens of chemotherapy**	1.537	0.836-2.823	0.166	1.664	0.856-3.235	0.134

**SLA (m mol/L) (≥4.0)**	1.873	0.864-4.061	0.112	2.058	0.881-4.807	0.096

**S-LDH (m mol/L) (≥225)**	2.001	1.038-3.859	**0.038** **∗**	2.481	1.226-5.019	**0.011** **∗**
**LDH≥225 and SLA≥4.0**	3.711	1.258-10.947	**0.018** **∗**	3.944	1.449-10.733	**0.007** **∗**
**Albumin (g/L) (≥40)**	0.388	0.216-0.697	**0.002** **∗**	0.384	0.205-0.721	**0.003** **∗**
**TP (g/L) (<70)**	0.717	0.403-1.278	0.260	0.967	0.518-1.807	0.917
**CEA (u g/L) (≥5.0)**	1.268	0.722-2.228	0.409	1.078	0.578-2.007	0.814
**CA 199 (U/ml) (≥40.0) **	2.233	1.219-4.091	**0.009** **∗**	2.201	1.155-4.196	**0.017** **∗**

*∗* We define P < 0.05 as statistical difference.

**Table 4:** Cox multivariate analysis of patients with metastatic colorectal cancer (N=57).

**Prognostic factors**	**PFS**			**OS**		
	**HR**	**95**%**CI**	**P value**	**HR**	**95**%**CI**	**P value**
**Locations (Rectum)**	**—**	**—**	**—**	0.359	0.174-0.740	**0.006** **∗**
**Pathological type (Poorly)**	**—**	**—**	**—**	—	—	**—**
**SLA (m mol/L) (≥4.0)**	**—**	**—**	**—**	**—**	**—**	**—**
**LDH (m mol/L) (≥225)**	**—**	**—**	**—**	2.204	1.000-4.858	**0.050** **∗**
**Albumin (g/L) (≥40)**	0.417	0.230-0.754	**0.004** **∗**	0.459	0.236-0.892	**0.022** **∗**
**CA 199 (U/ml) (≥40.0)**	2.072	1.125-3.816	**0.019** **∗**	**—**	**—**	**—**
**LDH≥225 and SLA≥4.0**	2.922	0.971-8.793	**0.056**	3.187	1.019-9.970	**0.046** **∗**

*∗*We define P < 0.05 as statistical difference.

## Data Availability

The data used to support the findings of this study are available from the corresponding author upon request.
